# Optimization of air supply mode of vehicle air conditioning based on digital thermal manikin

**DOI:** 10.1371/journal.pone.0339599

**Published:** 2025-12-29

**Authors:** Hongmin Xu, Rui Wang, Fengge Ma, Jing Xuan, Chaoqing Feng

**Affiliations:** 1 School of Energy and Power Engineering, Inner Mongolia University of Technology, Huhhot, China; 2 China National Institute of Standardization, Beijing, China; Southwest Jiaotong University, CHINA

## Abstract

With the rapid development of the automobile industry, the public’s demand for automobile diversification is growing steadily.People are no longer satisfied with basic driving convenience, more attention is now directed to the thermal comfort of the cabin. In order to build the relationship between in-vehicle environment parameters and thermal comfort index, the concept of equivalent temperature (Teq) was introduced, and then the DTM reflecting the thermal comfort of the human body was applied to the simulation of ANSYS Fluent. For a specific working condition of the vehicle air conditioning, the equivalent temperature of the in-vehicle environment was tested by the DTM, and it was found that the highest Teq (approximately 27°C) was observed in the foot region of the passenger. The Teq of most areas of the passenger’s body was relatively low, especially in the temperature sensitive torso, it was about 15–20°C. Numerical calculations for the same working conditions were carried out using the DTM. By comparing the results of Teq between the DTM and the physical thermal manikin, it was found that the results are very similar, the Teq deviation was less than 3℃, indicating that DTM can be used to measure Teq instead of physical thermal manikin. Finally, the DTM model was used to optimize the air outlet of the vehicle air conditioning system. The results showed that the best comfort was achieved when the air velocity was 4 m/s, the outlet temperature was 25℃, and the airflow was directed toward the occupant’s abdomen.This study demonstrates that DTM can replace physical experiments in thermal comfort evaluation, providing an efficient and low-cost approach for optimizing vehicle HVAC design.

## Introduction

With growing automotive demand and diversified user expectations, attention to cabin thermal comfort has intensified. According to ASHRAE, thermal comfort is defined as the state of mind that expresses satisfaction with the surrounding thermal environment. It results from the thermal balance between the human body and the environment [1,2].Thermal comfort is influenced by both environmental parameters (e.g., temperature, humidity, airflow, mean radiant temperature) and human factors (e.g., clothing insulation, metabolic rate) [3].Therefore, relying solely on ambient temperature fails to accurately assess human thermal comfort due to the combined influence of radiation, airflow, and humidity. To better align the thermal environment with comfort indicators, the concept of equivalent temperature (Teq) was introduced. Teq refers to the uniform ambient temperature in a hypothetical space where the mean radiant temperature equals the air temperature and the air velocity is zero. Under such conditions, the convective and radiative heat exchange (i.e., sensible heat exchange) between the human body and the surrounding environment is equivalent to that in the real scenario under evaluation [4]. Therefore, Teq enables accurate prediction of occupants’ thermal sensation using fewer environmental parameters.

Subjective thermal experiments are significantly affected by individual differences, leading to poor repeatability and reliability. When sample sizes are small, it becomes difficult to ensure the accuracy and generalizability of the results. Therefore, many institutions had developed and employed thermal manikins [5–7] to conduct experiments in automotive wind tunnel environments for evaluating vehicular thermal comfort. Feng et al. used thermal manikins to measure local and whole-body equivalent temperatures at the front passenger seat and predicted both global and segmental thermal sensations. The correlation between Teq-predicted thermal sensation and subjective results reached 0.911, verifying the accuracy of the proposed thermal comfort evaluation method for adult Chinese males [8]. Zoubayre et al. assessed thermal responses of frail individuals using a thermal manikin in a test cabin, comparing skin and core temperatures of males and females, and found significant time-dependent differences in thermal sensation [9]. Cernei et al. studied the effect of clothing insulation on predicted thermal comfort using thermal manikins [10]. Zhao et al. investigated human heat loss under varying sweating conditions with sweating manikins and found that moisture content and coverage of clothing significantly influenced thermal loss [11]. Del et al. employed sweating manikins to evaluate thermal physiological responses in ventilated short-sleeved jackets under warm-dry conditions [12]. However, the high cost and structural inflexibility of physical manikins and test equipment impose substantial burdens, particularly for research and small enterprises. This has led to widespread adoption of CFD-based numerical methods for analyzing vehicle cabin environments [13–15], enabling the use of DTM as substitutes for physical ones in thermal comfort studies. A DTM is a computer-based virtual model designed to simulate the heat transfer and thermoregulatory processes between the human body and its surrounding environment. It incorporates detailed human geometry, segmented physiological regions, and thermophysical properties, and is typically coupled with CFD to predict surface temperature distribution, heat flux, and thermal comfort indices. Unlike physical thermal manikins, a DTM allows flexible adjustment of posture, clothing, and environmental conditions in a virtual environment, enabling quantitative and visual analysis of human thermal responses in complex thermal scenarios. Ma et al. developed a CFD-based human body model incorporating clothing layers, analyzing heat loss under different environmental temperatures and validating results against experiments [16]. Hasama et al. combined experiments and CFD to study airflow characteristics from ceiling fans around heated DTMs and the resulting convective heat transfer, showing similar heat exchange in standing and seated postures, though with different contributions from average speed and turbulence [17]. Xu et al. applied a CFD-validated human-environment interface model to simulate the effects of wind speed, turbulence, and wind direction on convective heat transfer coefficients of the human surface [18]. Liang et al. used a DTM to evaluate the performance of a novel low-temperature radiant structure (LTRS), capable of improving local environments and overall indoor thermal comfort [19]. While computational simulation is widely applied in thermal comfort research, DTM has become particularly prominent in vehicle studies. Karthick et al. constructed the DTMs to assess the impact of different air vent types on thermal comfort in SUVs [20]. Ozeki et al. compared experimental and simulated Teq values in summer (with solar radiation) and winter (without radiation) using DTMs [21]. Zhang et al. used CFD simulations to examine the effects of seat heating and ventilation under extreme hot and cold conditions and developed a simulation framework that incorporates human-seat heat transfer for comfort evaluation [22]. Nesrine et al. validated airflow patterns from vertical ventilation systems using both thermal and digital thermal manikins, showing good agreement between simulations and experiments [23]. Zubieda et al. modeled heat transfer using a DTM head in a cavity to simulate steady-state dry heat loss with CFD [24]. Zasimova et al. assessed CFD prediction uncertainty of thermal comfort due to the DTM shape in displacement ventilation rooms [25], and also simulated 3D turbulent flow and heat transfer around seated DTMs in mixed ventilation rooms [26]. Abid et al. numerically evaluated the effect of inlet area on airflow and comfort while maintaining constant flow rate around the DTMs in a prototype ventilation chamber [27]. Pan et al. created a numerical model to predict thermal loss in sleeping DTMs and verified it experimentally [28].

This study adopted numerical simulation as an efficient approach that reduces experimental costs and effort while achieving accurate results. To further validate this method, the CFD-based Teq was compared with measurements from a physical thermal manikin. Compared to previous studies, this work introduced three key innovations. First, unlike previous CFD studies that primarily rely on air temperature or PMV as evaluation metrics, our study introduced Teq as the core indicator. This aligned with ISO 14505−2 and provided a more physiologically relevant evaluation of thermal comfort under non-uniform, transient vehicle cabin environments. Second, both CFD simulations and physical experiments were conducted with segmented evaluation, providing detailed Teq distributions across different body parts. Third, a multi-parameter optimization of air supply strategy was performed—considering air temperature, velocity, and direction—with Teq used to identify the optimal configuration, which effectively enhanced localized comfort while aligning with principle of “cool head,warm feet.” The study has verified the feasibility of digital simulation and providing a foundation for optimization strategies.

## Materials and methods

### Simulation using digital manikin

Utilizing CFD technology, this study performed numerical simulations to evaluate the thermal comfort of occupants in a vehicle cabin. Teq is chosen as the primary indicator for assessing thermal comfort. Unlike simple ambient temperature, Teq reflects perceived temperature influenced by external factors such as airflow and solar radiation, which create differences between actual and perceived temperatures. Teq accounts for conductive, convective, radiative, and solar heat exchanges impacting human perception, making it a comprehensive measure for both whole-body and localized thermal comfort.The DTM control model was designed to simulate the physical human body and includes three operational modes. In this study, the constant-temperature mode was applied, in which the surface temperature of the DTM was maintained at a constant 34℃ during simulation validation. In CFD simulations, DTM models are often configured with a constant surface temperature as an approximate boundary condition representing heat exchange between the human body and the environment [29]. Similar constant-temperature or “isothermal skin” settings have been widely applied in both virtual and physical manikin studies for comfort evaluation and benchmark validation, and are commonly used for CFD-based heat transfer analysis. Therefore, in this study, the human body surface was modeled with a constant-temperature boundary condition. However, thermal comfort is not determined solely by body surface temperature, but rather by the variations in surrounding environmental parameters such as air temperature, air velocity, and radiation. These changes alter the heat flux at the body surface. Teq is directly associated with surface heat flux. Hence, by keeping the body surface at a constant temperature and evaluating the changes in heat flux, Teq can accurately reflect the variations in thermal sensation.

#### Modeling and meshing.

**Fig 1:**
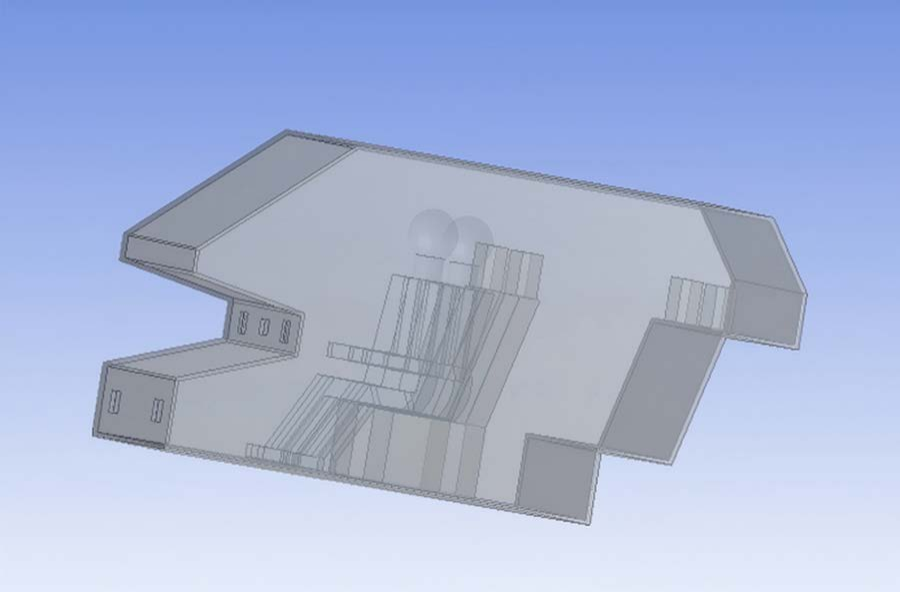
3D model of the vehicle cabin.

The vehicle cabin model used in this study is shown in Fig 1, constructed at a 1:1 scale. The boundary conditions and dimensions closely match those of the actual experimental setup. Complex surface geometries often lead to poor mesh quality and numerical instability during mesh generation. To avoid compromising accuracy, the vehicle cabin model was appropriately simplified replacing highly complex surfaces such as the dashboard with flat surfaces.These simplifications were limited to geometric features with little relevance to human heat exchange, the airflow distribution around the DTM was not affected, and thus the heat flux distribution and overall simulation accuracy remained unchanged. Therefore, the model simplification does not compromise the reliability of the results.Additionally, a digital driver model was added to the cabin to analyze the distribution of heat flux across the driver’s body surface. The thermal sensation of the driver was evaluated using the aforementioned method, thereby informing improvements in air distribution strategies for the air conditioning system.

Mesh generation was conducted using the Mesh module. Considering the complex internal structure of the cabin, unstructured tetrahedral meshes were employed. Since the study focuses on the heat flux and Teq of the human body, mesh refinement was applied to the DTM surface and air outlet regions (local size 3 mm, growth rate 1.2; with six boundary layers, transition ratio 0.272, and growth rate 1.2), while the rest of the cabin used default mesh to control the overall scale (cell sizes ranging from 15 to 30 mm). The final model contained approximately 2.1 million cells with an average quality of 0.84. This refinement allowed accurate resolution of local temperature gradients and small-scale flow structures, while maintaining a reasonable number of cells and avoiding excessive computational cost. Grid independence was verified by generating four sets of meshes with element counts of approximately 520,000, 1.02 million, 2.1 million, and 3.06 million. The simulations were conducted under identical conditions with the average surface temperature and the average convective heat-transfer coefficient of the body surface as the verification criteria, and the corresponding results are presented in Table 1. These comparisons demonstrate negligible sensitivity to mesh density, confirming the independence of the solution from mesh resolution. Although the average temperature and average heat transfer coefficient were already stable with the coarse mesh, local regions such as the DTM surface and air outlets could not be accurately resolved with a coarse grid. Therefore, a mesh with 2.1 million cells was selected, which ensured overall computational efficiency while better capturing local temperature variations and small-scale vortices, thereby improving the accuracy of the results. The generated mesh had an average quality of 0.84, indicating good mesh quality.

**Table 1:** Grid Independence Verification.

Number of Elements(×10⁶)	Average Temperature	Average Convective Heat Transfer Coefficient of the body surface
0.52	300.56K	2.91W/m^2^·K
1.02	300.76K	2.91W/m^2^·K
2.1	300.28K	2.92W/m^2^·K
3.06	300.58K	2.92W/m^2^·K

#### Boundary conditions.

The materials used in the vehicle cabin include plastics, leather, and similar substances, which exhibit low thermal conductivity and thus have limited heat conduction effects. Additionally, the hottest regions inside the cabin are relatively sparse, and interior materials generally possess low emissivity, making radiative heat transfer a minor component. Consequently, convective heat transfer plays the dominant role in thermal exchange within the cabin environment. To ensure consistency with experimental validation, the simulation boundary conditions were aligned with those used in wind tunnel testing.

[1] Physical Properties: The thermophysical properties of the main components in the cabin were defined based on literature relevant to in-vehicle airflow.
[2] Wall Boundary Conditions: The vehicle body was set as an opaque surface with a density of 8030 kg/m³, specific heat capacity of 502.48 J/(kg·K), thermal conductivity of 7.65 W/(m·K), absorptivity of 0.58, reflectivity of 0.42, and transmissivity of 0. Glass surfaces were modeled as semi-transparent with a density of 2529.6 kg/m³, specific heat capacity of 754 J/(kg·K), thermal conductivity of 0.91 W/(m·K), absorptivity of 0.4, reflectivity of 0.1, and transmissivity of 0.5. All surfaces were assigned mixed boundary conditions. The body surface was set to a constant temperature of 34℃. Seat surfaces were treated as adiabatic walls. The ambient air temperature was 30℃. Based on Stefan–Boltzmann law, the external vehicle wall temperature was estimated at approximately 60℃. The convective heat transfer coefficient was 28.8 W/(m²·K) for the vehicle body and 6.38 W/(m²·K) for glass.
[3] Inlet Conditions: Three velocity inlets were defined, each using turbulence intensity and hydraulic diameter settings. The velocity was set to 5 m/s for all inlets, reflecting the third fan speed in typical automotive air conditioning systems and aligning with experimental conditions.
[4] Outlet Conditions: Two pressure outlets were defined, with turbulence parameters matched to experimental settings. The outlet pressure was set to ambient atmospheric pressure, and outlet temperature was set at 30℃.
[5] Other Settings: The RNG k-ε turbulence model was used. The SIMPLE algorithm was employed for pressure-velocity coupling. Second-order upwind schemes were used for momentum, turbulent kinetic energy, turbulence dissipation, and energy equations. The DO radiation model was selected to account for solar effects, with solar irradiance set at 1000 W/m², simulating vertical incidence.

#### Results and analysis.

**Fig 2:**
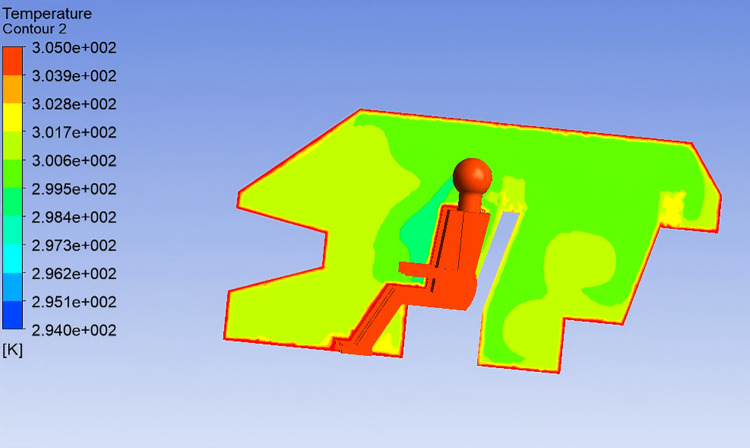
Cross-sectional Temperature Distribution.

After completing the CFD simulation in ANSYS Fluent, post-processing was conducted using dedicated software to analyze temperature and velocity distributions near the occupant. To facilitate this analysis, observation cross-sections were established, as shown in Fig 2.

As illustrated, the air temperature around the human body shows a vertical gradient: lower at the head and higher at the feet, consistent with the principle of ‘cool head and warm feet.“This phenomenon is attributed to the low airflow velocity in the lower cabin, where heat dissipation is limited, resulting in heat accumulation and elevated temperatures. The right forearm of the driver and the left forearm of the front passenger are largely exposed to the central air outlet jets, leading to the lowest observed temperatures in these regions. Overall, most of the cross-sectional area maintains a temperature range of 29–32℃. The highest temperatures are found in corners not reached by airflow, while the lowest, around 26℃, appear in front of the chest and face. The leg regions exhibit higher temperatures of approximately 31℃.

**Fig 3:**
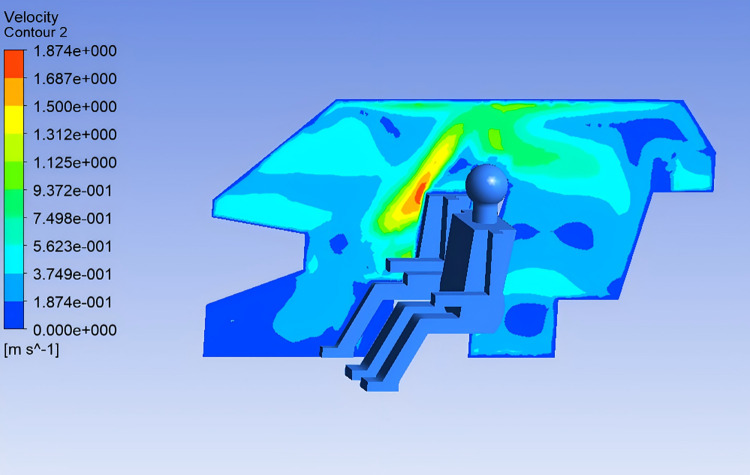
Cross-sectional Velocity Distribution.

As shown in Fig 3, the airflow velocity is highest in front of the occupant’s face and chest, reaching up to 1.6 m/s, which provides strong cooling under hot-summer conditions. The airflow around the legs and feet is moderate, ranging from 0.1 to 0.5 m/s, which helps maintain thermal comfort in the lower body.

#### Evaluation of thermal comfort based on simulation.

Due to the multiple heat sources in a vehicle cabin and the large gradients in temperature and velocity, the thermal environment is highly non-uniform. Therefore, equivalent temperature (Teq) was selected as the index for evaluating thermal comfort. Teq is calculated using [4]:


$$T_{eq}=T_{s}+\frac{Q_{t}}{h_{cal}}\;$$ (1)

${\text{T}}_{\text{s}}$: surface temperature (℃);

${\text{Q}}_{\text{t}}$: total convective radiation heat transfer(W/m²);

${\text{h}}_{\text{cal}}$: Comprehensive sensible heat transfer coefficient under a defined standard environment(W/(m²·K)).

The core idea of this formula is that the heat flux $Q_{t}$ (accounting for both convective and radiative components) measured in a calibrated environment corresponds to an equivalent temperature increment ΔT, which is added to $T_{s}$ to obtain the equivalent temperature.

**Fig 4:**
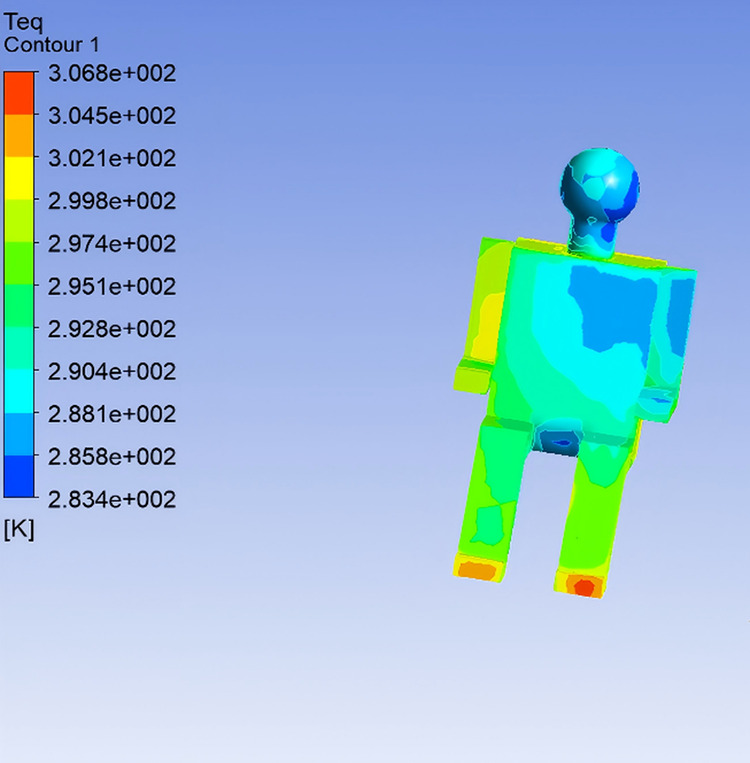
Equivalent Temperature Distribution of the Human Body.

In the simulation, the digital human model was operated in constant temperature mode, maintaining a skin surface temperature of 34℃. The body was divided into 13 segments: head, chest (front), back, left upper arm, left forearm, right upper arm, right forearm, left thigh, right thigh, left calf, right calf, left foot, and right foot. Heat flux for each body segment was extracted from post-processing, from which the surface Teq for each region was further calculated.As both the total heat transfer coefficient and heat flux on each body segment were available from the simulation, Teq were computed by incorporating clothing thermal resistance. The distribution of equivalent temperatures for the front passenger is shown in Fig 4 [2].

Teq for the left arm, chest, and head are relatively low, which correlates with the strong influence of the air vents. In contrast, the feet and right arm are less affected by the air outlet and are surrounded by warmer ambient conditions, resulting in higher Teq values. This observation aligns well with real-world expectations. The segmental equivalent temperatures of the human body are presented in Table 2.

**Table 2:** Equivalent Temperature of Each Body Segment.

Body Segment	Equivalent Temperature (℃)
Head	17.2366
Chest (Front)	18.0662
Back	27.5567
Left Upper Arm	15.5650
Left Forearm	16.6459
Right Upper Arm	26.0272
Right Forearm	25.0081
Left Thigh	19.0637
Left Calf	22.2889
Right Thigh	20.9506
Right Calf	21.8407
Left Foot	27.3589
Right Foot	26.2252

From this table, it can be seen that the lowest Teq occurs at the left arm, while the highest appears at the feet. These results are closely related to airflow velocity. Regions around the chest and arms experience the highest wind speeds and lowest air temperatures, resulting in lower Teq due to enhanced convective heat loss. Conversely, the feet are exposed to the lowest airflow and highest local temperatures, resulting in reduced heat dissipation and thus higher equivalent temperatures.

### Experimental validation

**Fig 5:**
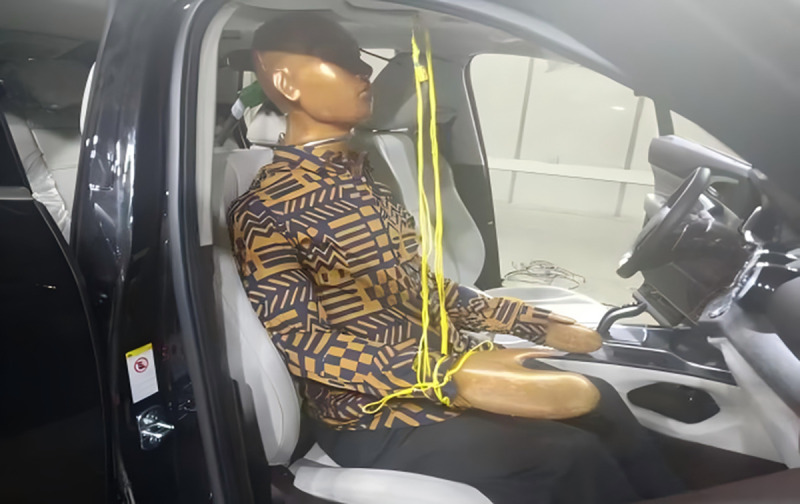
Experimental Setup.

To validate the accuracy of the simulation model, experimental testing was performed using a thermal manikin under specific conditions. A corresponding CFD simulation was conducted using the DTM, and the results were compared.The thermal manikin used in the experiment was life-sized and capable of whole-body heating. Based on the human thermal balance equation, the body was divided into multiple independently controlled heating zones, allowing surface temperatures in each region to closely match those of a real human subject. This setup enabled reliable representation of thermal sensation and comfort under different environmental conditions. Separate summer and winter conditions were defined to identify comfortable Teq ranges for various body segments. The test environment was constructed in an automotive wind tunnel laboratory. The ambient temperature was set to 30℃, with 50% relative humidity. Solar radiation was simulated as vertical noon sunlight at 1000 W/m². A vehicle speed of 100 km/h was simulated using the wind tunnel. The test focused on the front passenger seat of a specific vehicle model. The air conditioning system was configured to cooling mode at 22℃, with automatic fan speed, and airflow direction preset to a comfortable position based on prior subjective experience. Once environmental conditions stabilized, the thermal manikin was positioned in the front passenger seat. The experimental setup is shown in Fig 5.

After starting the engine and turning on the air conditioning, the vehicle was allowed to run for a period until both the car and its interior thermal environment stabilized. During testing, the thermal manikin was dressed to simulate typical summer clothing, including a long-sleeve shirt, vest, long trousers, underwear, cotton socks, and leather shoes. The total thermal resistance of the clothes was 0.7 clo.

**Fig 6:**
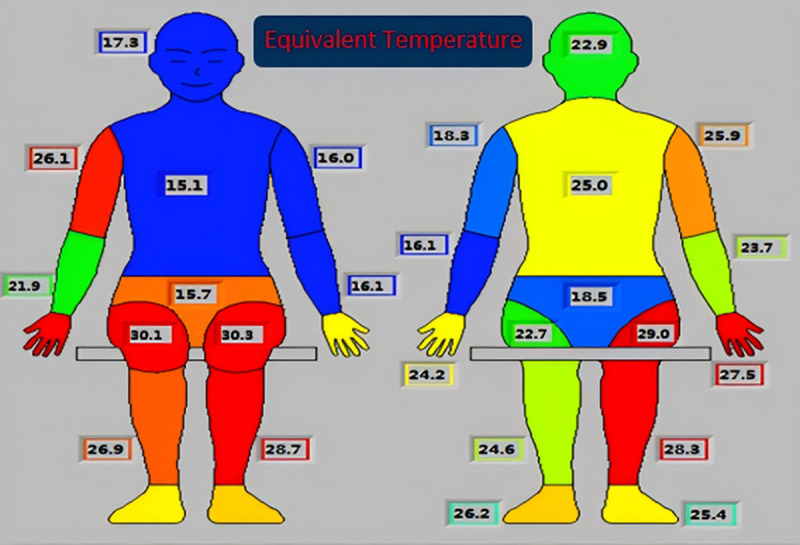
Equivalent Temperature Distribution in Experimental Conditions.

Once the air conditioning was activated, the interior temperature gradually decreased over time, and the spatial temperature distribution became increasingly non-uniform—forming a typical non-uniform thermal environment. The thermal manikin was used to measure the Teq corresponding to different parts of the body in this environment. After the manikin reached thermal equilibrium, the control software displayed the Teq across various body regions, as shown in Fig 6.

**Fig 7:**
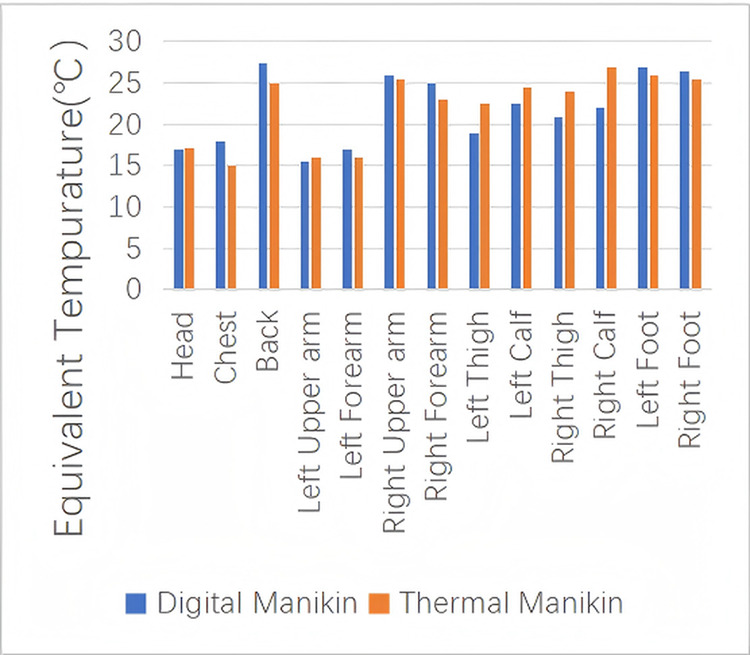
Comparison of Experimental and Simulated Teq Values.

The test results showed that the highest Teq were found on the front of both thighs and the rear of the right thigh. These were followed by relatively high Teq on the right hand, right calf, and front side of the right upper arm. In contrast, the lowest Teq were observed on the left arm, chest, and face. By applying area-weighted averaging to the Teq of all body parts, the whole-body Teq was calculated as 23.1℃. After computing the Teq for 13 body segments in the simulation using the DTM, a segment-by-segment comparison was conducted with the corresponding experimental values measured using the physical thermal manikin, as shown in Fig 7.

The figure illustrates the comparison results of Teq. Due to differences in the DTM’s placement posture and slight deviations in airflow direction during simulation, there are some discrepancies between the experimental and simulated results.To evaluate the accuracy of the simulation model in predicting the Teq of each body part, this study adopted three common error metrics: Mean Absolute Error (MAE), Root Mean Square Error (RMSE) and Pearson correlation coefficient (r). These indicators help to assess the difference between simulation results and experimental data. The errors for each region are calculated using the following equations:


$$\text{MAE}=\frac{\text{1}}{\text{n}}\sum_{\text{i}=\text{1}}^{\text{n}}\left|{\text{T}}_{\text{sim},\text{i}}-{\text{T}}_{\text{exp},\text{i}}\right|$$ (2)

$$\text{RMSE}=\sqrt{\frac{\text{1}}{\text{n}}\sum_{\text{i}=\text{1}}^{\text{n}}{\left({\text{T}}_{\text{sim},\text{i}}{-\text{T}}_{\text{exp},\text{i}}\right)}^{\text{2}}}$$ (3)

$$\text{r}=\frac{\sum_{\text{i}=\text{1}}^{\text{n}}\left({\text{T}}_{\text{sim},\text{i}}{-\text{T}}_{\text{sim}}\right)\left({\text{T}}_{\text{exp},\text{i}}{-\text{T}}_{\text{exp}}\right)}{\sqrt{\sum_{\text{i}=\text{1}}^{\text{n}}{\left({\text{T}}_{\text{sim},\text{i}}{-\text{T}}_{\text{sim}}\right)}^{\text{2}}}\cdot \sqrt{\sum_{\text{i}=\text{1}}^{\text{n}}{\left({\text{T}}_{\text{exp},\text{i}}{-\text{T}}_{\text{exp}}\right)}^{\text{2}}}}$$ (4)

${\text{T}}_{\text{sim},\text{i}}$: Simulated temperature of the number i part

${\text{T}}_{\text{exp},\text{i}}$: Test temperature of the number i part

${\text{T}}_{\text{sim}}$: Average simulated temperature

$T_{\text{exp}}$: Average experimental temperature

n: Total number of parts (13 in this study)

The mean absolute error (MAE) was 2.93°C, and the root mean square error (RMSE) was 3.99°C. The model achieved high accuracy in most regions. Larger deviations were mainly observed in the leg area, where the simulated values were lower than the experimental results. This difference may be attributed to the fact that in the experiment, the legs were in contact with the seat, allowing heat to be slowly transferred from the seat to the legs, while in the simulation the seat was modeled as an adiabatic wall without heat feedback, leading to a lower surface temperature. Nevertheless, the Pearson correlation coefficient between the two datasets was 0.65, with a p-value of 0.016 (p < 0.05), indicating a significant correlation between the simulated and experimental results. Overall, the errors were acceptable, and the model agreed well with the experimental data, demonstrating reliable performance in predicting whole-body thermal responses.These findings demonstrate that the DTM can effectively replace the thermal manikin for evaluating thermal comfort in non-uniform environments, thus providing theoretical support for further cabin comfort optimization using simulation techniques.

## Results

### Airflow optimization using DTM

The three key factors affecting automotive HVAC comfort are air outlet temperature, airspeed, and airflow direction. To assess their individual effects, one parameter was varied while the others were held constant, and the resulting thermal comfort was evaluated using Teq as the metric. The six working conditions and their respective air supply settings are summarized in Table 3.The airflow condition in Condition 1 corresponds to the simulation setup described in the previous section and was adopted as the baseline experimental condition for comparative analysis against the other scenarios.

**Table 3:** Boundary Conditions for Different Working Conditions.

Condition	Air Supply Temperature (°C)	Airspeed (m/s)	Airflow Direction for Front Passenger
1	22	5	Toward chest and left arm
2	25	5	Toward chest and left arm
3	28	5	Toward chest and left arm
4	22	4	Toward chest and left arm
5	22	6	Toward chest and left arm
6	22	5	Toward right abdomen and right arm

#### Analysis of the influence of supply air temperature.

**Fig 8:**
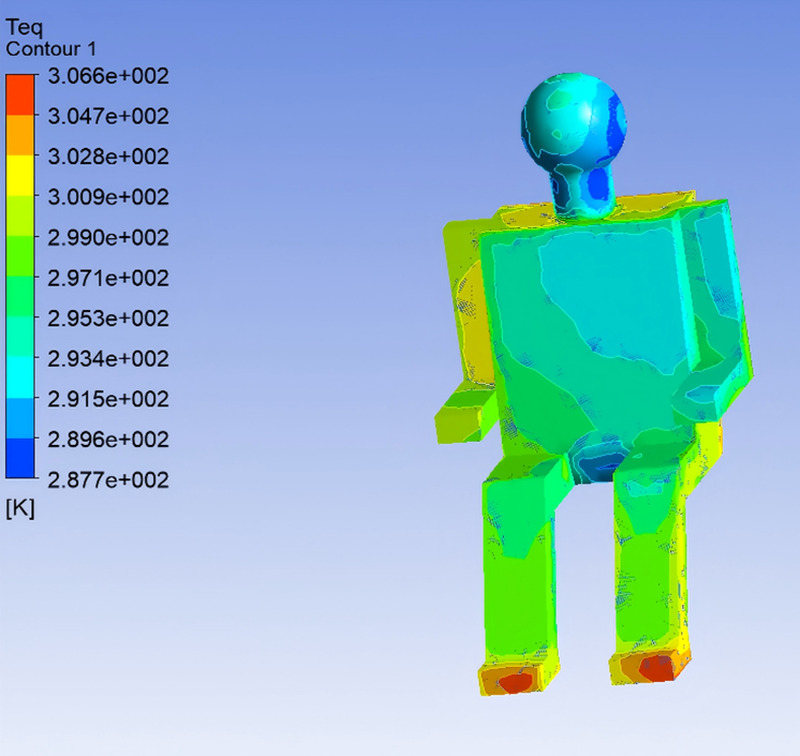
Equivalent Temperature Distribution under Condition 2.

**Fig 9:**
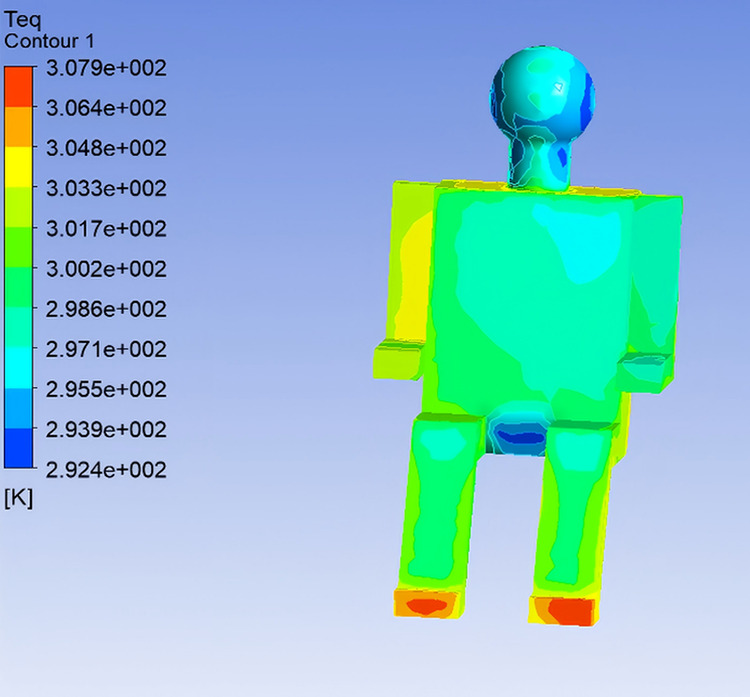
Equivalent Temperature Distribution under Condition 3.

To investigate the influence of supply air temperature on human thermal comfort within the vehicle cabin, the airflow velocity was maintained at 5 m/s, and three supply air temperatures—22℃, 25℃, and 28℃—were selected for simulation analysis, corresponding to Conditions 1, 2, and 3, respectively. The following section presents an analysis of how variations in supply air temperature affect Teq of the human body and the perceived thermal sensation. Based on the simulation results of Conditions 2 and 3, the distributions of Teq across different body segments were determined using the same methodology, as illustrated in Figs 8 and 9.

The analysis of Teq distributions under different conditions reveals that, although the distribution trends across body segments remain largely unchanged with increasing outlet air temperature, the overall thermal sensation tends to shift toward a warmer state. In Condition 1, the relatively low outlet temperature results in noticeably cooler sensations in the chest and left arm regions. As the outlet temperature increases, the Teq rises in Conditions 2 and 3, leading to a sensation of overheating in some body regions.

#### Analysis of the influence of airflow velocity.

**Fig 10:**
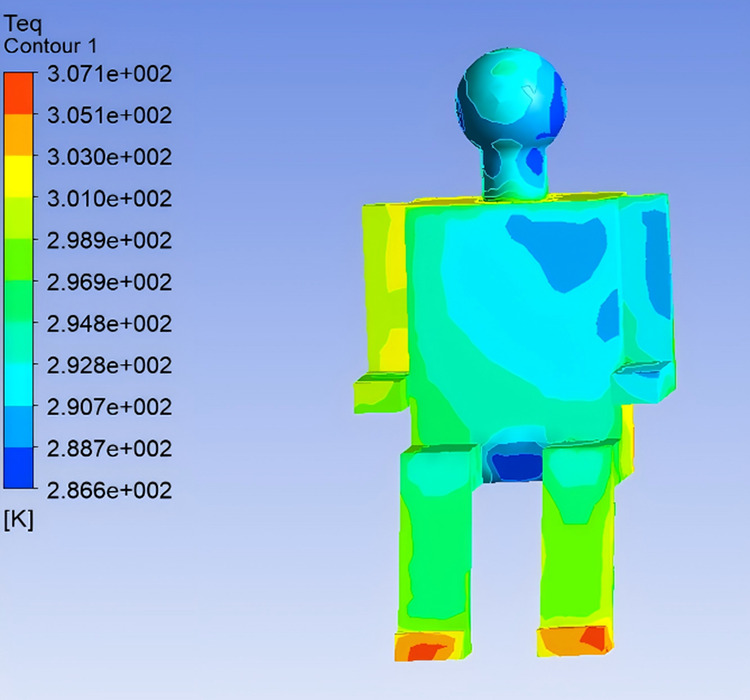
Equivalent Temperature Distribution under Condition 4.

**Fig 11:**
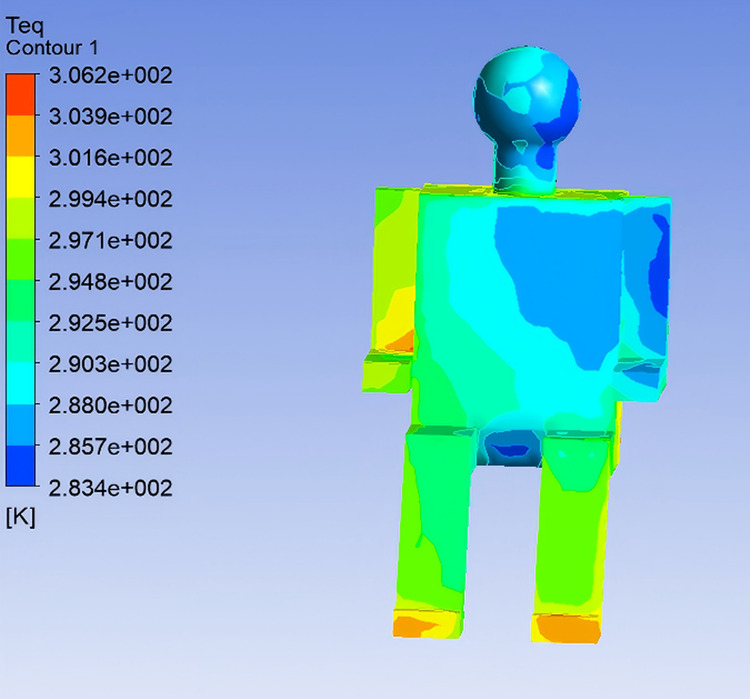
Equivalent Temperature Distribution under Condition 5.

To investigate the effect of airflow velocity on thermal comfort within the vehicle cabin, the supply air temperature was kept constant at 22℃, while three airflow velocities—4 m/s, 5 m/s, and 6 m/s—were selected for simulation analysis, corresponding to Conditions 4, 1, and 5, respectively. The influence of airflow velocity on Teq and thermal sensation of the human body were examined in this section. By analyzing the simulation results for Conditions 4 and 5 (excluding the previously discussed Condition 1), the Teq distributions for various body segments were obtained using the same method, as illustrated in Figs 10 and 11.

Analysis of Teq distributions under different airflow velocity conditions reveals that, as the supply air velocity increases, the Teq across various body segments generally tends to decrease. Notably, body regions directly exposed to the airflow exhibit a more rapid drop in Teq. When the airflow velocity exceeds 5 m/s, Teq at non-directly-impacted regions tend to stabilize. In Condition 5, the high airflow velocity results in significantly lower Teq values in the chest and left arm regions, indicating a cooler sensation. In contrast, Condition 4, characterized by a lower airflow velocity, yields relatively higher Teq values in the same regions, indicating improved thermal comfort.

#### Analysis of the influence of airflow direction.

**Fig 12:**
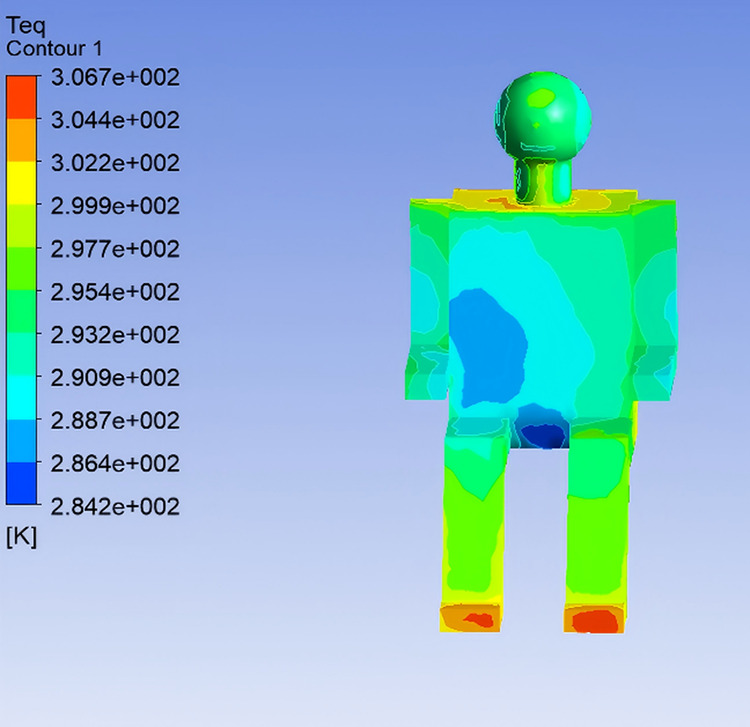
Equivalent Temperature Distribution under Condition 6.

Based on the analyses in the previous two sections, it was observed that variations in temperature and airflow velocity primarily altered the numerical values of temperature and velocity at different cabin locations, without significantly affecting the overall distribution of the flow field within the passenger compartment. Although such changes can improve thermal comfort to some extent, they typically result in increased energy consumption. Therefore, enhancing thermal comfort by modifying the airflow direction alone, while keeping temperature and velocity constant, represents an important research direction.To examine the impact of airflow direction on in-cabin thermal comfort, the outlet temperature was maintained at 22℃, and the airflow velocity at 5 m/s. Given that the head region in Condition 1 was already perceived as cold, only two airflow directions were considered for simulation: toward the chest and left arm (Condition 1) and toward the right abdomen and right arm (Condition 6). The resulting changes in Teq and thermal sensation across different body segments were analyzed. Using the same methodology, the segmental Teq distribution under Condition 6 is shown in Fig 12, providing a basis for comparison with Condition 1.

From the analysis of Teq distributions under Conditions 1 and 6, it can be observed that variations in airflow direction lead to noticeable shifts in the Teq distribution across body segments. The regions closer to the adjusted airflow angle experience the most significant changes in Teq, while regions farther from the modified airflow path show relatively minor variations. Specifically, in Condition 6, a larger surface area of the chest is exposed to direct airflow, resulting in a lower Teq in that region compared to Condition 1.

#### Optimized model.

**Fig 13:**
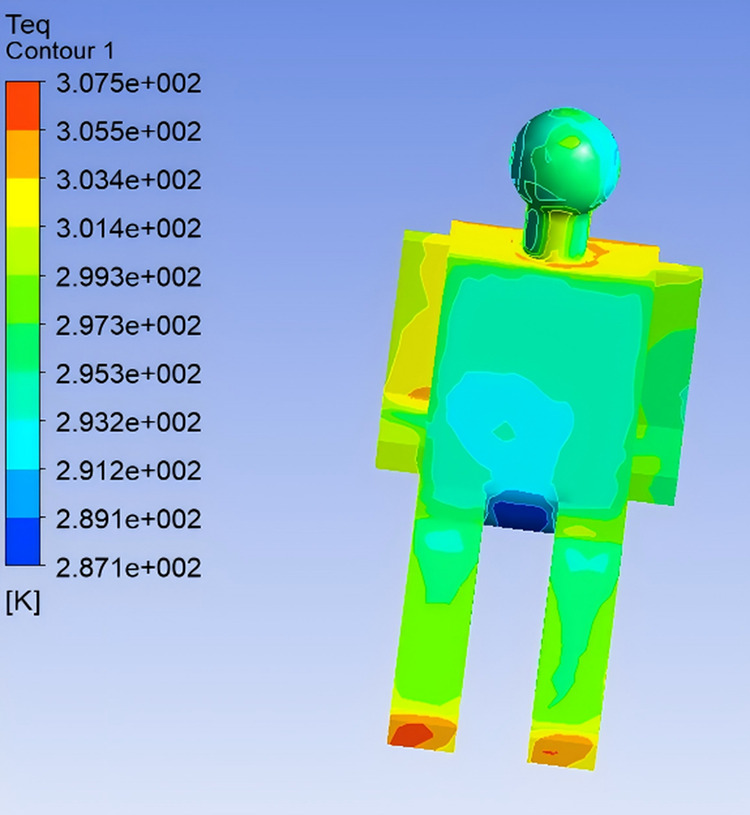
Equivalent Temperature Distribution After Optimization.

By comprehensively evaluating the effects of the three influencing factors and comparing Teq distributions across all working conditions, the air outlet configuration was optimized. The optimal condition was identified as: reducing the airspeed to 4 m/s, setting the air outlet temperature to 25℃, and adjusting the airflow direction to target the mid-abdomen. All other boundary conditions remained consistent with those used in the previous simulation validation, and the geometric structure and dimensions of the model were unchanged. Under this configuration, the equivalent temperature distribution across all body segments was recalculated using the same simulation methodology, and the results are illustrated in Fig 13.

**Fig 14:**
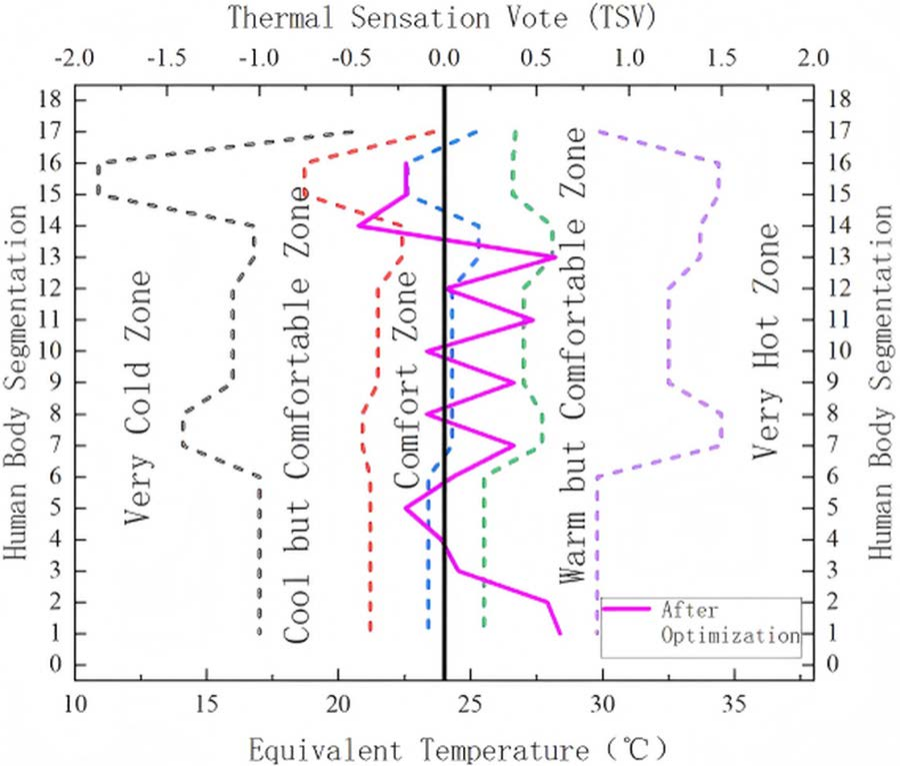
Evaluation Results of Equivalent Temperature After Optimization. (1: Right Foot, 2: Left Foot, 3: Right Calf, 4: Left Calf, 5: Right Thigh, 6: Left Thigh, 7: Right Forearm, 8: Left Forearm, 9: Right Upper Arm, 10: Left Upper Arm, 11: Upper Back, 12: Chest, 13: Face, 14: Head, 15: Overall).

With the adjusted airflow direction, most body regions experienced noticeable airflow, resulting in a more uniform distribution of equivalent temperature. To maintain the ergonomic principle of “cool head and warm feet,” the optimized airflow direction deliberately avoided direct flow to the feet, preserving higher Teq in that region. Prior to optimization, body surface temperatures were generally too low. By simultaneously lowering the airspeed and raising the outlet temperature, the optimized configuration shifted all body regions closer to the thermal comfort zone. The localized Teq under this condition were validated against the summer thermal comfort evaluation zone specified in international standards [2], as shown in Fig 14.

As a result of the increased air supply temperature and reduced airspeed, the Teq of all body segments increased, bringing them closer to the thermal comfort range. Moreover, due to the adjustment in airflow direction, the Teq varied across body regions, but all remained within the internationally defined comfort zone.

## Conclusion

In this study, both digital and physical thermal manikins were used to simulate the thermal comfort in a vehicle cabin. Equivalent temperature (Teq) was employed to evaluate thermal comfort across various regions of the human body, leading to the following conclusions:

[1] Simulation with the DTM revealed that the highest Teq (~27℃) appeared at the feet of front-row passengers, while most other body parts—especially temperature-sensitive torso regions—had lower Teq values ranging from 15–20℃. Rear seat regions showed weaker airflow exposure and relatively higher ambient temperatures.
[2] Using the DTM to optimize the cabin’s air supply configuration, the optimal condition was determined as: airspeed of 4 m/s, air outlet temperature of 25℃, and airflow direction towards the abdomen. Under this condition, the driver’s thermal comfort was optimal.
[3] Experimental testing with the thermal manikin yielded Teq for different body segments. Comparison with simulations showed an average deviation of less than 3℃, within acceptable error range, confirming the reliability and applicability of the proposed simulation model.
[4] The optimization process based on Teq effectively improved the distribution of thermal comfort throughout the human body. After optimization, the perceived thermal comfort of the occupant was significantly enhanced and more aligned with standard comfort zones.

### Nomenclature

**Table:** 

CFD	Computational Fluid Dynamics	DTM	Digital Thermal Manikin
DO	Discrete Ordinates radiation model	HVAC	Heating, Ventilation, and Air Conditioning
Teq	Equivalent temperature(℃)		

## Supporting information

S1 Table Minimal data set. (XLSX)
